# Selections and Abstracts

**Published:** 1900-08

**Authors:** 


### *SELECTIONS AND ABSTRACTS.*

---

### Some of the Causes of Pains in the Feet.*

Pains in the feet are very frequently encountered, and personally I have many times been at a loss to know how to interpret them. The numerous articulations, with their synovial membranes, the complicated ligamentous and fascial structures, the bursas, the arteries, and the nerves all come in for their share in the production of painful affections. The functions of the foot as an organ of support and progression also expose it to numerous injuries and mechanical alterations. In addition to the local conditions we must consider general and constitutional diseases which are important factors in the causation of pain.

It is with the idea of grouping together at least some of these causes, of giving the leading facts in regard to the diagnosis, and of calling attention to some of the less common affections, that I have prepared this paper. It is not my object to discuss affections or injuries associated with marked and conspicuous anatomic changes, such as the common fractures, tumors, tuberculosis, etc., but rather to consider affections not accompanied by very evident changes of form. For convenience, a rough classification has been attempted, and we begin with certain inflammatory affections.

Acute, subacute and chronic articular rheumatism of the articulations occur, of course. The chronic rheumatic arthritis, or *arthritis deformans*, attacks particularly the smaller joints—acute exacerbations of the process are associated with pain. To these rheumatic inflammations and to gout I shall only refer in passing. It is to gonorrheal inflammations that I wish to allude more in detail.

The various forms of so-called gonorrheal rheumatism frequently involve parts of the foot, and the pains produced are often very persistent and resist attempts at cure. Tendon sheaths, joints, bursas and fibrous tissue may be affected, and the manifestations of

---

*Read by C. A. Hamann, M.D., Cleveland, Professor of Anatomy, Medical Department Western Reserve University, before the Cleveland Medical Society, February 23, 1900.

the process vary greatly as regards clinical signs and duration. The subdivision of gonorrheal arthritis into the acute and chronic forms is not very satisfactory, inasmuch as most cases begin acutely but have a chronic course. Gonorrheal rheumatism may be classified into monarticular and polyarticular forms. Koenig, from observations on a large number of cases in the Charité in Berlin, makes a classification into four groups: 1. *hydrops articuli.* 2. Serofibrinous inflammation. 3. Empyema of the joint. 4. Phlegmonous inflammation.

When the metatarsal joints are involved it is hard to determine which ones are the seat of the disease, owing to the somewhat diffuse swelling of the soft parts. Indeed, it is likely that several joints are affected, for they are close together and communicate, though not to the same extent as in the wrist; in the foot, as well as in the hand, the numerous tendon sheaths are often involved and give rise to certain peculiarities as contrasted with gonorrheal arthritis of the knee. The pain in the acute stages is great, as a rule, and there is considerable inflammatory infiltration of the soft parts. The course is apt to be protracted and exacerbations occur from time to time. I have not seen suppuration occur. In the wrist I have met with suppuration and extensive destruction of the carpal bones, necessitating resection of the joint.

The pain may be due to gonorrheal fascitis, or an inflammation of the fibrous tissues and bursas about the heel. These heel pains are not uncommon in gonorrheal rheumatism. In the following case is illustrated a somewhat typical form of gonorrheal rheumatism, in which the bursa between the tendo Achillis and the upper part of the tuberosity of the os calcis, together with the fibrous tissues about the os calcis, were the seat of inflammation:

A. H., aged 19, had gonorrhea in July, 1898, and two attacks since then. About one month later he began to have pain in the right heel. This was followed by pain in the left heel. Standing aggravated the discomfort, and he was unable for part of the time to continue his work as a machinist. The urethral discharge was cured, and all sorts of remedial measures were resorted to for the relief of his heel pains. Protracted rest, blistering, antirheumatics, steel sole-plates, and the use of the hot-air appliance alike failed to relieve him.

When I saw him, about a year after the beginning of his trouble, the following condition was found: Both heels, especially the right, were enlarged; posteriorly upon the sides there was thickening of the fibrous structures and a trace of edema. Upon pressure over the insertion of the tendo Achillis there was considerable pain, also pain on pressure over the under-surface of the heel. There was no evidence of flat foot. No joints were involved. As about all remedial measures had been exhausted, I suggested to him the advisability of opening the bursas, curetting and draining. This was done, with only partial relief, however. At the operation there was found some turbid serum in the bursas. Cultures from the fluid failed to show any micro-organism.

The term Achillodynia was applied by Albert to cases in which there was pain upon pressure over the insertion of the tendo Achillis, without apparent cause. It is now known that the causes of this pain are to be sought in an inflammation of the bursa retrocalcanea, which may be due to trauma—either a sudden one or repeated friction, as by badly fitting shoes—to gonorrhea (as in the case above mentioned), to syphilis, to gout, to rheumatism (rarely), or to tuberculosis. In some instances no cause can be definitely determined.

Fournier and Jacquet claim that gonorrheal periostitis and osteitis of the os calcis are quite frequent, and they have referred to the condition as the "*pied blenorrhagique.*" It is much more likely, however, that it is primarily an involvement of the bursa and a secondary thickening and infiltration of the fibrous tissue and periosteum.

Ledderhose has described a peculiar form of plantar fascitis, which at times is painful, and occurs as a result of nutritive disturbances in patients who are confined to bed with fractures or other injuries of the lower extremities. Fibrous nodules at times form in the fascia, and these are painful.

The "painful subcutaneous tubercle" of the old writers is also met with in the sole of the foot.

Francke describes a number of cases of fascitis due to influenza; also joint and periosteal inflammations, all of which give rise to pains in the feet. In a more recent article he states that many of these cases, however, are really plantar neuritis. The possibility of arthritic, periosteal and osseous inflammation being due to typhoid infection is also to be borne in mind.

The various forms of neuritis, traumatic, toxic, etc., may occur in the plantar nerves. I have seen, for instance, a punctured wound in the sole of the foot followed by a severe neuritis of the internal plantar and posterior tibial nerves. Post-typhoidal neuritis of the plantar nerves may occur. Hughes has seen a severe form of plantar neuralgia in caisson-disease.

In neuritis the pain is generally of a burning character, and the patient complains of various paresthesia. The diagnosis of plantar neuritis will be based largely upon tenderness of the nerve-trunks when pressure is made over them. The lightning pains of locomotor ataxia can only be referred to in passing.

Flat-foot: This is perhaps the most common cause of pains in the feet and a cause that is often overlooked. It is important to examine carefully the plantar arch in all cases of painful feet. The pain in these cases may be present from the beginning—or it may appear after a twist or a sprain of the previously diseased foot. The pain is not necessarily commensurate with the degree of deformity, mild cases being often associated with considerable suffering. At first there is usually a sense of fatigue, followed after a time by aching; these uncomfortable sensations frequently extend to the calf of the leg, and even to the thigh. As we would expect, exercise increases the discomfort, and rest relieves it. If the condition is unrelieved and the patient persists in standing on his feet, the pain becomes more severe. It exists during the night time, and when the patient awakes in the morning he finds that there is stiffness of the feet.

As the result of overexertion, sprains, and inflammatory conditions of the articulations, the pain may be temporarily increased. There is in most cases certain points which are tender upon pressure. One of these is over the inferior calcaneonavicular ligament which supports the head of the astragalus and is subjected to stretching in flat-foot; in front of and below the internal malleolus, anterior to the tibiotarsal articulation, and at the bases of the metatarsal bones, are other painful points. Rotating or twisting the foot inwards I have found usually increases the pain.

One frequently finds pain in the feet associated with mild degrees of *pes planus*, and varicose veins of the leg, particularly during and after middle life in women who have become corpulent.

In persons who have been confined to bed for some time I have frequently observed pains in the feet to follow when they first get up. Particularly is this the case if the patient has to walk with crutches as the result of an injury of one limb. The sound limb then has to support the entire weight of the body, the arch of the foot has a tendency to sink down and what we might call acute flat-foot develops. This is, however, a temporary condition and usually disappears soon. The affection described by French writers as "policeman's disease" is probably a manifestation of flat-foot.

Plantar hyperesthesia may be due to badly-fitting, tight and thin-soled shoes.

The pain in flat-foot is due to the stretching of fibrous, ligamentous and muscular structures, to the contact of parts of the bones not normally in apposition to others, and to inflammatory changes in the articulations.

In recent years there has been considerable discussion among German military surgeons regarding a peculiar form of painful swelling of the foot, seen in soldiers. Not to weary you with details, it may be stated that the condition is due to a partial fracture of one of the metatarsal bones—usually the second or third. The fracture is caused by indirect force and occurs in soldiers who are compelled to make forced marches over rough ground, or who suffer from contusions, twists and falls. There is a painful swelling on the dorsal aspect of a metatarsal bone and examination with the Röntgen rays and the formation of a callus show that a fracture has occurred.

When the leg and foot has been confined in improper positions in the treatment of injuries, such as fractures, there often remain painful conditions of the feet and ankles. In these cases the deformity, as for instance after a Pott's fracture, chronic inflammatory process, thickening of fibrous tissues, deposits of callus, and the imprisonment of nerve-fibers, account for symptoms observed. The affection first described by T. G. Morton, and known as metatarsalgia or Morton's disease of the toes, is characterized by pain in the vicinity of the fourth metatarsophalangeal articulation, and by the fact that pressure over the joint and lateral compression of the metatarsus increase the pain. At times the patient is unable to wear a shoe. The disease is much more often encountered in women than in men. It is believed by Morton and others that the pain is caused by the compression of branches of the external plantar nerve between the heads of the fourth and fifth metatarsal bones.

Vascular disturbances: Changes in the arteries, both in the large as well as the small ones, are responsible for various pains in the feet and legs.

Many cases of senile gangrene, due to thrombosis, embolism or arteritis, are preceded for a time by uncomfortable sensations, paresthesias and burning pains in the toes. Jonathan Hutchinson also calls attention to cases of "arterial anesthesia" in which, owing to vascular change, there is at intervals numbness of the feet. In these cases, the sensory disturbances are probably due to defective nutrition of the nerves.

Erb has recently called attention to cases of "intermittent limping" in which endarteritis, involving the smaller arteries is the causative factor. Charcot in 1858 first described this condition as due to an obliteration of large arteries. Veterinary surgeons had observed it in horses before this time.

The clinical picture, as represented by Erb, is characteristic: while the patient is at rest he is quite comfortable; after walking for five, ten, thirty minutes, various sensory, motor and vasomotor disturbances appear—such as itching, crawling sensations, a feeling of coldness, tension of the skin, pain, pallor, cramps, stiffness and difficulty in walking. The feet, calves of the legs and thighs are the seat of these abnormalities, which may reach such a high grade that the sufferer is unable to walk or stand. In some cases there sooner or later appear inflammatory changes in the toes and feet—ulcers form, and gangrene may ensue. Erb seeks to show that these disturbances are due to *endarteritis obliterans*, involving the smaller arteries of the leg and foot. He lays particular emphasis on the disappearance of the pulse in the anterior and posterior tibial arteries as diagnostic of the affection, and insists on a careful examination of these vessels. Erb has shown that by early recognition of the condition and appropriate treatment, much can be done to relieve the patient and defer the onset of the severer symptoms.

Manteuffel calls attention to similar cases of angiosclerotic gangrene, occurring in younger persons; the main symptoms are the severe pains in the feet. In connection with the above cases, Raynaud's disease and erythromelalgia may be referred to, in which, as well known, there are vasomotor disturbances.

Referred or reflex pains: Pain in the heel has been noticed by many observers in association with urethral stricture; also by von Pitha in a case of vesical calculus. Removal of the stone completely relieved the patient. Renal calculus, inflammation of the neck of the bladder and of the prostate, and neuralgia of the bladder, so-called, may also cause this reflected or referred pain in the heel. The celebrated surgeon, von Pitha, himself suffered from this.

Reflex pains in the feet are sometimes observed in cases of uterine and ovarian diseases.

It unfortunately is the case that in some instances it is impossible to find the cause of the pain, much less to relieve it, and patients go from physician to physician in vain attempts to be cured.—*Cleveland Journal of Medicine.*

---

### LABORATORY AND PERCENTAGE FEEDING OF INFANTS IN HEALTH AND DISEASE.*

Those who have accustomed themselves to percentage feeding will never wish to employ any other method. Yet misconceptions arise among those ignorant of the proceeding regarding the real nature of it. A contrast of the old and the new methods may tend to do away with these.

In the old way physicians made empirical mixtures of certain

*Abstract of paper by J. P. Crozer Griffith, M.D., read May 23, 1900.

numbers of ounces of cream, milk, water, and so on, without any accurate knowledge of just what the final result consisted in or without knowing the reasons for the changes made. In fact, much further progress was impossible, owing to the variability in the strength and purity of the milk and cream employed.

All we could do was to shift numbers of tablespoonfuls up and down as the needs of the child seemed to demand.

With the newer plan physicians simply adopt the percentage analyses of milk and cream which have been furnished by investigators, and apply these to practical feeding. By this method they do their thinking and make their calculations in the elemental percentages of the ingredients—that is to say, mixtures are now composed of this or that *percentage* of fat, proteids, etc., instead of so many *tablespoonfuls* of milk and cream and water. This method is more scientific, and the results more satisfactory. The calculations are simple, and several easy formulæ have already been published.

But to work in percentage feeding it is necessary to have milk and cream of a definite percentage strength. We may assume that sugar and proteid of ordinary herd milk varies but little. It is the percentage of fat which is the variable quantity. Any physician can, however, readily test the fat strength with the simple centrifuge sold for general medical purposes, and can then calculate his mixture accordingly. When cream and milk of a definite strength in fat can be obtained, even the simple home testing is obviated.

This brings us to a consideration of milk of certified strength, as furnished by several different dairies in this country, and especially to the question of milk laboratories as managed by the Walker-Gordon Company in various cities. The purpose of a milk laboratory is not always understood. It is merely a place where you can order anything you want in the way of food for an infant—even proprietary foods if you wish them. To speak of a baby being fed on "Walker-Gordon milk" shows misconception. There is nothing special about this milk, except that its strength in fat and proteid is guaranteed, and that especial care is taken for its purity. You may mix it up in any way you please, just as with any other milk. The Walker-Gordon milk and cream may be purchased and the mixture made at home according to the directions given by the physician.

But even by this method the calculation of a formula by the physician is necessary, and the mother has a lot of work to do. With a milk laboratory handy, all this trouble can be avoided by having the mixtures made at the laboratory according to prescriptions furnished by the physician. In this case he merely specifies in the prescription the desired percentages of fat, proteid and sugar wanted, the number of bottles for twenty-four hours and the amount in each, and so on. Anything whatever that is desired may be prescribed—barley water, egg water, pancreatin, etc.

There has been some little talked and written against milk laboratories on account of the use of "separator" products. But all dairy cream nowadays is separator cream. The objection therefore is trivial.

Finally it is necessary in laboratory feeding, as in all feeding, to know what to expect from the different ingredients, and what proportions are likely to be required. As a rule, every very young infant, and older ones with feeble digestion, should be started with low percentages; say 0.50 or 0.66 of proteid instead of the normal 1 or 1.50 of human milk, and with the fat, and possibly the sugar as well, lower than normal. This is because we are dealing with a milk other than human. If curds are passed in large quantities the proteids are probably too high. If there is much rancid vomiting the fat is probably in excess. The low percentages are to be increased as soon as possible; but there should be absolutely no fixed rule about a positive increasing of percentage strength to keep pace with increase in the months of life. No such increase occurs in human milk. The proper gain in body weight should be the chief guide. I may say in passing that I have never found the slightest difficulty in increasing a proteid percentage with laboratory milk more than with any other milk, as has been claimed by some physicians. I cannot conceive of any reason for such a difficulty, nor do I believe that it exists. It is merely necessary that we increase the percentages of the ingredients in the proper way, when the need for increase comes.

(The details of three cases were then given, with wall charts, illustrating this point. Each child had been fed previously more or less on laboratory prescription milk. Each was in wretched condition when seen. Each gained in an entirely satisfactory way after the proper percentages had been found. The fact was emphasized that a hasty judgment would have condemned laboratory feeding in these cases. Yet it was not the laboratory feeding but the physician's prescriptions which had been at fault.)—*Philadelphia County Medical Society Proceedings.*

---

### THE TREATMENT OF SCARLET FEVER.

This subject was discussed at some length in the section of Diseases of Children at the annual meeting of the American Medical Association (reported in the Philadelphia *Medical Journal,* July 1), and Dr. R. A. Birdwood, medical superintendent of the Park Hospital, Lewisham, has stated his experience in the *Hospital,* April 15, 1899. In an ordinary mild uncomplicated attack of scarlet fever Birdwood keeps the child in bed for three weeks on a low diet with stewed fruit till three days after the temperature has fallen to normal. The bowels are opened daily, and the urine tested for albumin on alternate days. The reason for so long a stay in bed is that if nephritis supervenes it usually does so about the end of the third week, and the maintenance of the regular action of the bowels seems to have a marked and beneficial effect in preventing edematous swelling of the legs. They are also detained in bed whilst albuminuria or dilated heart persists, and when in bed are blanket-bathed daily. It is not prudent to use the bath during this time, as faintness or a bad color comes on occasionally. During convalescence, if all goes well, a warm soap-and-water bath is given three times weekly. As a rule, this is sufficient treatment for desquamation. A. Gilbert recommends anointing the skin with lard, vaseline or lanolin. If desquamation continues, a weak acid solution, such as an ounce of dilute acetic acid to half a pint of water, applied to the soles or palms on lint for a quarter of an hour, or else rubbing with glycerine and borax, does much to remove adherent epithelium. Desquamation may go on for three or four months, or even longer, and it is quite common for it to come on twice and sometimes three times.

*High Temperature.*—B. Gilbert objects to the use of coal-tar antipyretics, and to the full bath unless the fever is very high and there is very active delirium. The nervous excitement can best be allayed by chloral hydrate. Slagle and Ewing agree in objecting to antipyretic drugs, but advocate the free use of cold water for drinks, enemas and packs. By drinking water freely they contend that the toxins are eliminated. Quayle prescribes lithia water and Ewing prescribes potassium acetate in addition. Garrison has used acetanilid for eight years, and finds it useful; it promotes a feeling of well-being, and if combined with soda bicarbonate does not diminish the renal secretion. Garrison does not hesitate to employ antipyrin if the pulse denotes high arterial tension, and has given it to young infants in doses of a quarter of a grain. Birdwood finds that the high temperature of scarlet fever is well controlled by sponging with tepid water, and the patient generally feels better after it is done. It is good practice to sponge whenever the four hourly temperature exceeds 39°C. Hyperpyrexia of scarlet fever is not controlled by baths or drugs; the former may be frequently repeated, but the temperature rises again, and the patient becomes rapidly worse. Now and again a patient recovers after a temperature of 41° C., and this encourages one to keep on with tepid sponging. An undoubtedly good effect is sometimes observed in the reduction of temperature on removal of blankets, and leaving the patient covered with a sheet only. If either sponging or removal of blankets induces shivering or a feeling of coldness or actual coldness of the extremities, it should be discontinued, and warm water bottles should be used. A thirsty fever patient should be given plenty of drink, and it is well to remember that there is sometimes a repugnance to milk. Water relieves thirst best, and should be given. Grape or orange juice is generally liked, and does good. Barley water is a suitable drink.

*Sore Throat.*—A chlorine gargle is used at the Park Hospital (chlorate of potash, ℥iss.; hydrochloric acid, ʒvj., with five pints of water added after the evolution of the gas) when an acid preparation is desired, or liq. sodæ chlorinatæ 1 in 15 of water when an alkaline. Equal parts of either of these chlorine solutions and warm water are mixed just before use. If the patient is not old enough to gargle, a ball syringe with a long nozzle attached is used for flushing out the fauces. Two 4 oz. syringefuls are enough. Should the patient resist the attempt to pass the nozzle between the teeth, there is no occasion to use force to do so. The fauces can be well washed by passing the nozzle between the cheek and the teeth, so that its point goes just beyond the last molar tooth. This should be done first on one side, then on the other. The patient should be held with the face downwards. The practice of gargling the throat is far better than swabbing, for the tissues are often soft enough to be damaged by the latter proceeding.

*Nose and Ear Discharges.*—A solution of boric acid (℥ii. to 5 pints) is used by Birdwood; before use it is mixed with an equal quantity of warm water.

*Nephritis.*—Birdwood usually orders loin poultices or fomentations. In some instances the drawing of a few ounces of blood has been followed by a flow of urine; in others, no such result has ensued. Seidlitz powders or compound liquorice powder should be given, not calomel. Solomon prescribes cascara and sodium phosphate, and also a decoction of scoparius (℥i. to Oi) to relieve the kidney congestion.

*Joint and Muscle Pains.*—Solomon has found acetanilid useful in relieving the severe headache and pains in joints, but where muscular pain was complained of he gave phenacetin instead. Birdwood employs salicylate of soda.—*Dr. Francis J. Allan's Abstract in Treatment, September 14, 1899, Canada Medical Record.*

---

### Does "Cross-Eye" Affect the General Health?

A. L. Ranney,* New York, reports four cases of squint which are particularly interesting examples of what can be done toward restoration of single vision by modern methods. The summary and conclusions reached after a discussion of these cases are as follows: (1) A small percentage of subjects in whom cross-eye exists, even to a degree of extreme deformity, owe their disfigurement

** N. Y. Medical Record*, July 21, 1900.

entirely to errors of refraction. Proper glasses alone will correct the disfigurement of such patients. Extreme nervous phenomena may coexist in this type of case with the cross-eye, and disappear entirely when the refractive correction is properly made by glasses. (2) Extreme and constant disfigurement from cross-eye, which does not prove to be the result of refractive errors, does not as a rule entail eye-strain or tend to create reflex nervous disturbances. (3) Those who suffer only occasionally from cross-eye, and at other times show no cast, are peculiarly liable to reflex nervous diseases. These subjects are constantly on the border-line of double vision, and are wasting nervous force incessantly in their unconscious endeavors to maintain binocular vision. The red-glass test, and the exclusion tests, are of great diagnostic value with such patients. (4) Extreme cross-eye, inward or outward, is occasionally due to the fact that both eyes are adjusted either too high or too low in the orbit. This is a clinical fact that has been unrecognized by oculists until of late. It is a most important point to decide by the aid of the tropometer prior to operative interference in all cases of lateral squint. (5) Some subjects are unconsciously able to adjust for very high degrees of "latent" squint, and actually to maintain binocular single vision most of the time. The red-glass test in such cases usually develops unconquerable diplopia at once. Severe nervous troubles are very common in patients of this type. The extreme maladjustment of the eye-muscles is very apt to be overlooked and remain uncorrected. This is because the squint is not constant, and when present is too often attributed solely to physical debility, excessive use of the eyes, etc. (6) The phorometer and tropometer are often essential to the proper recognition of the causes of cross-eye, and the particular muscles at fault in individual cases. (7) To those who have not one of these instruments, the employment of the "Maddox rod" and the red-glass will sometimes furnish extremely valuable information regarding the causation of reflex nervous disturbances from eye-strain. Patients who are on the border-line of squint will very often reveal their existing eye-strain at once (in the form of unconquerable diplopia) when the red-glass test alone is made. (8) The "exclusion test" is of great value in cases of squint. It is often extremely difficult and sometimes almost impossible to teach patients afflicted with cross-eye to abandon their unconscious habit of suppressing visual images. This test then becomes the chief reliance of the oculist in determining both the form and degree of maladjustment that exists, and in many instances which operative step to take first. (9) When vertical and lateral squint coexist, it is usually wise to correct the vertical maladjustment, either entirely or in part, prior to operation upon the lateral muscles. Too much haste in operative procedures for the correction of combined vertical and lateral squint is apt to lead to unsatisfactory results. (10) The old methods employed in operating for cross-eye have been so modified since the discovery of cocain that the most severe deformities can be rectified to-day without pain, and even with no confinement to the house. (11) The author would impress upon his readers that a scientific correction of most cases of cross-eye takes more time than is usually allotted to such cases. It is vitally important to endeavor to get as exact and perfect an adjustment as possible, and to save the patient many annoyances and possibly a nervous breakdown that are apt to be entailed by imperfect operative work, and the consequent eye-strain in overcoming a tendency to diplopia that never before existed in the same degree.—*St. Louis Medical Record.*

---

### SHALL THE SPECIALIST DIVIDE THE FEE WITH THE GENERAL PRACTITIONER?*

When an attorney in a county seat has a client in danger of the penitentiary, and hence in need of the very best of counsel, it is customary for him to seek some eminent lawyer of a great city and request his aid. In so doing does he approach the distinguished gentleman and say: "I have a client accused of ——, who is able to pay $3,000 for his acquittal—will you take the case with me for this sum, leaving me the gratification of having done my professional duty?" By no means! He plainly states: "My patron

*Extract from paper read before the Missouri State Medical Society, May, 1900, by Emory Lanphear, M.D., Ph.D., St Louis, Mo., formerly Professor of Surgery in the Kansas City Medical College and the St. Louis College of Physicians and Surgeons; Gynecologist to St. Joseph's Sanatorium.

has $3,000 for his defense—are you willing to take $2,000 of this to join me in securing justice for him?"

Arrangements of this kind are made daily in every large city. Does any one ever suggest that the country attorney has been doing a dishonorable act in thus securing his brother practitioner to do the major part of the work for the $2,000, he retaining $1,000 for his services? Would a doctor sued for $100,000 regard such a transaction as disgraceful, unethical, objectionable, if thereby he were saved this sum?

But let the question be one of saving life instead of securing liberty or preventing financial loss, and how different it is!

If a country doctor have a patient with recurrent appendicitis (upon whom he *might* operate with success, but fears possible failure) with a prospective fee of $600, must he, in order to be "ethical," write to some city surgeon to come to his help, take all of the $600, and leave him merely the satisfaction of a duty well performed, or possibly pay for a few visits at starvation rates? "Upon what meat doth this our Cæsar feed that he hath grown so great?"

Why should not the country doctor plainly say to the city specialist: "I have a patient with appendicitis who is able to pay $600. Will you operate for $400 and leave me $200 for preparation, after-treatment, etc.?" What would be wrong about this? Let Drs. Robert T. Morris, of New York, and Burnside Foster, of S. Paul, who so vigorously maintain that division of the fee is unethical under any and all circumstances, point out what injustice would thereby be done to (a) the patient, (b) the attending physician and (c) the eminent surgeon. Why should we not learn a few things from the methods of our most noted lawyers, men who are above suspicion as to unethical conduct? Have we not hitherto been too unmindful of the financial interests of ourselves and our professional brothers?

I maintain that the payment of a "commission" for all business simply "referred" to a specialist, or for mere consultation, is probably unethical, certainly demoralizing in tendency; but that division of the fee is perfectly honorable and right when the specialist and the general practitioner jointly share the work and the responsibility.

### MERCUROL IN THE TREATMENT OF GONORRHEA.

At a meeting of the Genito-Urinary Section of the New York Academy of Medicine, held on the 21st of March, Dr. Ferd. C. Valentine reported a case of acute gonorrhea treated by mercurol irrigations. The patient was an American, aged 32, married, the secretary of a corporation; and was unusually anxious to get well with as little loss of time as possible. He had had several previous gonorrheas, resulting in stricture. On January 21 last, while inebriated, he had coitus extra domum. Three days afterwards he found a free yellowish discharge with the usual pain on urination. He at once put himself under treatment, and for ten days was irrigated regularly with mercurol, for a part of the time twice a day. Discharge was reduced from a free yellow flow to a slight pin-head drop by the first irrigation of mercurol, 5 per cent., and the urine became clear. Microscopic examination of a specimen of the discharge which was taken on the first day, showed numerous gonococci characteristically grouped in pus cells. Two days later, after the fifth irrigation, the gonococci were found to have disappeared. A burning sensation was experienced after the irrigations, but the strength of the solution being reduced, the pain gradually became less and ultimately ceased. While he did not present the case as absolute proof of the applicability of mercurol as a gonococcide, he thought the results obtained were sufficiently satisfactory to warrant further tests. The preparation, he added, was a new one, prepared by Dr. Karl Schwickerath of Detroit.

Dr. Ramon Guiteras said mercurol was being used at the New York Post-Graduate Hospital. The treatment was less drastic than that described by the reader of the paper, the custom at the institution referred to being to commence with small dosages and gradually increase their strength, especially when new preparations were being experimented with. In the case of mercurol they had commenced with as mild a solution as one-half per cent., and finding favorable though rather slow results, they had gradually increased it, until now a solution of two per cent. is given to all patients who present themselves at a clinic devoted exclusively to this mode of treatment, of which Dr. Otis K. Newell has special charge. He (Dr. Guiteras) was not sanguine about the discovery of a germicide which would cure gonorrhea in the brief time their unprofessional brethren with their remedies claimed to be able to do; but, on the other hand, he did not wish to be regarded as a pessimist, and if mercurol proved to be as much an improvement on protargol and argonin as they had done on permanganate and nitrate of silver, it proved at all events that they were progressing along the correct lines.

Further report of experiments in progress with mercurol are to be given at future meetings.

---

### MEDICO-LEGAL QUESTIONS RELATING TO LABOR, PREGNANCY, AND CONCEPTION.

Professor Brouardel (*Revue de Méd. légale, etc.*, May, 1900) discusses a number of topics of the above sort, the first relating to the possibility of unconscious impregnation and gestation.

He cites a number of cases. In one, a girl of weak intelligence, readily allowed herself to be seduced, and then related all the details of her escapade. This behavior was considered tantamount to imbecility, and the man in the case was visited with additional penalties for taking advantage of a feeble-minded girl.

In other cases the state of pregnancy was apparently ignored by the women until labor had set in; one woman even had no suspicion of her condition until the cries of the child led her to realize it. It is proper to state that this ignorance on the part of the woman was often due to erroneous diagnosis by the attending physician.

The next subject considered relates to impregnation during ordinary sleep. An inn-keeper's wife, sleeping profoundly after a hard day's work, felt merely a sense of weight upon her, which she really believed to be caused by her husband's presence. She was not conscious of any intermission within the vagina. However, she woke in time to see a man servant in the room arranging his clothing. He was convicted on her testimony. Crime of this sort is favored by overcrowding in tenements and small habitations.

In Russia, on account of the inclement weather, individuals of both sexes crowd into the same bed for the sake of warmth, and women are probably abused without their knowledge. In one instance two people, man and woman, became intoxicated. Neither had any recollection of sexual intercourse, yet in no other way was it possible to explain the pregnancy which followed.

The sleep induced by drugging plays a prominent part in cases of involuntary conception. Men often take advantage of inexperienced women in this manner. But there are certain fallacies here. Women often allege that they become unconscious immediately after a suspected potion has been drunk, and that half an hour or so later they are able to go about their work. Medical men know that this cannot be true. Not only is a considerable interval required for the drug to act, but after ingesting such a powerful substance a prolonged reaction accompanied by nausea, malaise, etc., would inevitably result.

An allied topic is the anesthetic sleep. The daily press once asserted that an organized band of ravishers, employing chloroform, infested the compartments of railway trains. Many researches have been made to ascertain whether or not a sleeping person can be chloroformed without wakening.

Dr. Berger obtained ten positive results out of twenty-nine experiments. However, it is not as much the woman as the doctor and dentist who are apt to suffer from abuses due to anesthetic sleep. The subject is, of course a trite one for the physician.

Brouardel next discusses the hypnotic sleep. Cases of alleged abuse during this type of unconsciousness are rare. The first upon record dates from 1858. The most celebrated case is probably the one in which Levy, a Parisian dentist, figured. He pretended that before proceeding with his work it was necessary to examine the genitals of a young girl. This was done without the girl or her mother having any suspicion that anything wrong had occurred. The girl became pregnant. It was found that Levy had been able to hypnotize women and ravish them without even attracting the attention of the mother or third person who was sitting near by while the supposed vaginal examination was being made. Levy was sentenced to ten years' imprisonment in 1878.—*Obstetrics.*

### THE DOCTOR'S INCOME.

The average income of a physician in large cities on this continent may be placed at $2,000, in the smaller towns at $1,500, and in the rural districts at $1,200. Two or three New York physicians are said to make over $100,000 a year, five or six about $50,-000, but the average income, although rather higher than in Chicago and in other American large cities, does not greatly exceed $2,000 yearly. The minister averages in this city perhaps $1,200, and in the country certainly not more than $800 yearly. As regards living expenses, both the lawyer and the minister have an advantage over their professional brother. In New York, for example, office accommodation suitable to a physician is very dear, in a good neighborhood costing not less than $70 to $80 a month, which, with board and lodging and other necessary disbursements, will represent a sum of $120 monthly—a sufficiently weighty burden for a struggling youthful practitioner to bear. The young minister has no rent to pay, while the legal neophyte can regulate his outlay in this respect according to the length of his purse. Nevertheless the lot of the medical beginner, compared with that of a pastor in a like situation, has its compensation. He is at least more or less independent. The minister, on the contrary, is, as a rule, permitted to exercise his own will but to a limited degree, and often is doomed to go through a lifetime of toil subservient to the caprices of censorious elders and deacons. An excellent description of the trials of an American country minister and the various unpleasantnesses with which he has to contend at the hands of his congregation is given in the "Damnation of Theron Ware," the best novel written by the late Harold Frederic. When all is said that can be said, the first few years of medical practice are years of arduous effort, full of disillusionment and disappointment. The late Sir Andrew Clarke told Dr. Osler: "From the vantage-ground of more than forty years of hard work he could say that he had striven ten years for bread, ten years for bread and butter, and twenty years for cake and ale." The truth undoubtedly is, and especially in the large centers of population in America, that the opportunities for a physician to obtain adequate compensation for his services are yearly becoming less. This is not due to any deterioration in the quality of the present-day practitioner or an evidence of falling off in medical or surgical skill. The fact is irrefutable that the medical profession in this and all civilized countries stands on a higher plane in the matter of training and knowledge than ever before. The reason for the decrease in medical incomes is indubitably almost wholly owing to the more eager competition among regular practitioners, to hospital and dispensary abuse, and to the lamentable increase in quackery. The supply of medical men is greater than the demand, the market is flooded; and the most potent remedy we can suggest for this evil is that, as has been many times advised in the *Medical Record*, a uniform high standard of medical education should be established in every State.—*Medical Record.*

---

### THE MEDICAL MISSIONARY.

The New York *Medical Journal* says that the Ecumenical Conference on Foreign Missions, which has roused a great deal of popular interest in the sessions it has been holding in New York, did a most praiseworthy and judicious thing when it decided to devote considerable time to medical missionary work. We fear it must be confessed that the importance of such work is more thoroughly appreciated by the clerical missionaries, if not, indeed, by ecclesiastics in general, than it is by members of the medical profession as a body. Many of the missionary clergy recognize that their wards' physical needs come quite as distinctly within their sphere of ministration as their spiritual instruction does, and they freely admit that every missionary ought to have some medical knowledge. One gentleman who spoke is reported to have declared that a missionary "must always be ready to undertake the cure of any disease brought to his attention." We shall assume that he said, or meant to say, *care* instead of cure—the types do play such pranks with public utterances—for we conceive that the pretense of power to cure all diseases might easily prejudice the cause among the heathen, many of whom are capable of reasoning quite astutely; but surely every missionary should be capable of alleviating physical suffering, if nothing more.

We wonder that physicians have heretofore shown so little interest in the work of the medical missionary, and our wonder grows when we reflect that the medical missionaries have not been mere plodders in relieving individual cases of disease, but have striven to keep up with the advance of medicine and have even themselves contributed to it in no mean measure. We have often had occasion to reproduce material published in the *China Medical Missionary Journal*, for example, and we never look over an issue of it without finding something that entitles it to a place in medical libraries as well as in evangelical esteem. Moreover, there are eminent medical men engaged in missionary medical schools, and in the mission work itself there is many a man who would have been sure to achieve distinction in medicine had he stayed at home. But we hope this conference will be found to have enlisted in behalf of the medical missionaries the earnest cooperation of the great body of his professional brethren, and we are encouraged in this hope by the fact that the President of the New York Academy of Medicine, and one of its ex-presidents, were among those who took part in the proceedings of the conference. Other eminent physicians, too, both American and foreign, have taken a prominent part in the good work, and we feel sure it will have its effect.

---

### HOW FAR IS THE SURGEON JUSTIFIED IN OPERATING ON HOPELESS CANCER CASES?

The *International Journal of Surgery* says that some of the saddest cases the surgeon is called to see are those in which cancerous disease has made such headway that no operation can offer even a reasonable hope of cure. The ideal surgery of cancer consists in early interference, with thorough removal of all tissues either infected or forming a possible nidus for further development of neoplasmic growth. It certainly is often an injustice to the patient to propose an operation that is not decidedly likely to at least prolong life for a reasonable period of time, and yet is not always proper to refuse all surgical interference, merely for the purpose of gathering favorable statistics. If an operation is able to relieve, even for a time only, the mental anguish and the physical suffering of such patients, it should be undertaken; not, however, without warning the friends and relatives, if not the patient himself, of the temporary nature of the improvement that is expected to follow. It is especially in the forms of carcinoma attended with much ulceration, sloughing and hemorrhage, that the surgeon is distinctly bound to interfere. Ligation of the lingual arteries in the digastric triangles, with removal by the galvano-caustic wire, of a stinking, sloughing, bleeding tongue, is a humane procedure that is apt to give the doomed patient many days of surcease from his pain. Thorough curetting of a cancerous uterus that has gone beyond the limits of hysterectomy, followed by cauterization with the Paquelin, or with chromic acid, and later by treatment with formaldehyde, or some other strong disinfectant, will often bring relief that is none the less welcome because it is but temporary. In all such conditions surgery is certainly capable of lessening suffering and of giving the patients a little of that hope which, although it is based on delusive grounds, so seldom utterly forsakes them. In visceral cancers, as in carcinoma of the pylorus, the disease often kills, more by mechanical effects than by distinct pathological alterations, and various forms of gastro-intestinal anastomoses are justified in some instances in which removal of the affected tissues is impossible. Since latter-day surgery has shown that the stomach is not indispensable for the maintenance of life, it is a question whether the establishment of duodenal or jejunal fistulæ, through which patients might be fed, is not indicated in some of these cases.

---

### Recent Opinions on General Paralysis of the Insane.

The analysis of current literature in the *Revue Neurologique*, January 30th, 1900, considers a number of articles recently published on this subject.

Verhoogen stated at the Congress of Insurance Examiners at Brussels that it behooved the medical staffs of life insurance companies to be especially on the lookout for this disease because of its growing frequency. The following symptoms might excite a warrantable suspicion: Facial paralysis, anisocoria, exaggerated patellar reflexes, reflex iridoplegia, tremor of facial muscles, troubles of speech and writing.

Roques and Fursac (*Thèse de Paris,* 1899, 140), who have studied under Joffroy, combat the theory of the syphilitic origin of this disease. They claim that most paretics are degenerates who exhibit certain stigmata. Analyses were undertaken of fifty normal men and fifty each insane and paretic. It was ascertained that the paretic presented the same stigmata as did the insane, and to the same extent. The predisposition is, therefore, held to be the same in both classes.

Campbell, at the last meeting of the British Medical Association, stated as a result of his investigations that sixty per cent. of paretic men were certainly syphilitic, while enough more were probably contaminated to bring the total up to eighty-seven per cent. As for the balance, they were in doubt, there being no evidence for or against infection. The same subject was discussed by the Société Médicale des Hopitaux, Paris, last November. Joffroy had seen paretics with lesions of tertiary syphilis. Specific treatment always cured the latter without influencing the former. He thought syphilis could cause paresis only indirectly, like alcohol or overwork. Serieux and Farnarier, the authors of the paper of the evening, found an incubation period of fourteen or fifteen years an average between date of primary syphilis and appearance of paralysis. The former disease is usually contracted about the age of twenty-five, while paresis develops about forty.

Mariani's thesis (Paris) was devoted to heredity in paretics. Of 33 cases there were in the ascendancy of 10 patients, insanity ; of 11, neuropathy; of 17, alcoholism, etc. In many cases there was an association of two or more of these taints in the same subject. Thus most cases of neuropathic inheritance were conjoined with alcoholism.

Macdonald, at the last meeting of the British Medical Association, reported several cases of idiot children presenting the symptoms and pathological findings of paresis as seen in the adult. Toulouse and Marehand also report a case in which general paralysis in a child simulated idiocy.—*Medical Review of Reviews.*

---

**This Journal and Atlanta Semi-weekly Journal one year for $1.00 cash in advance. No checks. Strictly to new subscribers.**

### THE TREATMENT OF RICKETS.

For patients in the first years of life, from one and one-half to five years, my plan of campaign is practically the same for all. For those with heavy bodies and heads, protuberant bellies, head sweats, and commencing or acquired bow-legs or knock-knees, the food supply is carefully attended to, the digestive functions restored as soon as possible, starchy food being limited, fats being recommended, especially bacon fat and drippings, which I believe has for these cases a high nutritive value. For very young children especially the addition of some cream to the dietary is most valuable. Condensed milk, if it be taken, should be replaced by cow's milk boiled, and a weak gravy soup or broth will often help to stop the rickety tendency. For marasmic patients nothing acts so well as a daily rubbing under each axilla of cod-liver oil. For all these rachitic patients, then, varying the dose slightly according to age, I prescribed cod-liver oil and syrup of the phosphates of iron. In very hot weather I let the children leave off the oil, but the mixture generally is well taken, and the results are most satisfactory. In cases with any tuberculous or syphilitic history, the syrup of the iodide of iron should be substituted for that of the phosphates. Next as to the treatment of the rickety deformities, especially of the leg bones. In the case of patients under the age of four years, even with very marked bowed and bent tibiæ, and some degree of knock-knee, I can assure the parents with great confidence that after nine months' to fifteen months' treatment these deformities will be very greatly improved or altogether remedied. I am prepared to leave any question of operation to a later age—say that of five or six years. By that time we can see whether ordinary treatment has been able to effect anything; the bones have got harder, and there is less risk of producing the calamitous condition of pseudarthrosis. These children's legs should be put up in splints, preferably outside ones, which may purposely be made three or four inches too long. The splints should be taken off once every month or three weeks in the winter and once a fortnight in the summer. I do not deny that a rickety child's legs occasionally become much straighter with no treatment whatever. Still I feel sure that the great mass of these rickety children are far better when off their feet—for this reason, that the deformity of the pelvis is so much less apt to occur, as little or no pressure is acting upwards through the acetabula while the child is lying down or even sitting up.—*Pediatrics.*

---

### PROPRIETY.

An unpleasing feature of the recent meeting of the American Medical Association was the distribution to all comers at the meeting place of the general sessions of printed copies of a paper upon "The Gynecologic Consideration of the Sexual Act," which had been read at the Columbus meeting and had been refused publication in the official journal. No doubt the author had no intent that the paper should fall into the hands of any one but the members, and no doubt he felt the necessity, in his attempt to enforce publication of his paper, of placing a copy in the hands of the members present so that they might act intelligently. Still it was his place to be careful as to the methods he employed, and the fact that copies fell into the hands of women and children of course determined the action of the president in ordering the distribution stopped, and undoubtedly influenced the vote that was registered later against permitting the publication of the paper in the Association's journal. Really more fuss arose over the matter than the occasion deserved. There was nothing in the paper that could do harm in the hands of physicians, and there was something that it might be worth while to know. Still the editor and trustees had a perfect right to refuse publication if they chose, and it was the Association's duty to stand by its officers. It is certainly true that a great deal of false sentiment was displayed in regard to the matter, and many held exaggerated views as to the effect of the publication of the paper, forgetting that frequently in these matters it is open opposition that gives the widest circulation. Undoubtedly the author, having expressed his intention of bringing his chosen subject prominently before the profession, attained his end much more readily by reason of the antagonism that he excited than he would if the paper had been treated in the ordinary manner and more or less ignored. So probably the author is best satisfied at the turn taken by events, while those who differed from him are gratified at their success in tabling his motion to force the trustees to publish the paper. No vote was taken upon the merits of the case.

Like other hobby-riders the author holds an exaggerated view of his subject, which, while of some real importance, did not justify the methods employed to enforce its publication. It is impossible to resist the temptation of referring to one remarkable error that occurs in the paper. The author speaks very confidently of the prevalence of "cunnilinguistic practices," meaning of course "cunnilingual," as the relation of "linguistics," or the science of language, to the first portion of the compound word would at best seem to be very obscure!—*Cleveland Journal of Medicine.*

---

**This Journal and Atlanta Semi-weekly Journal one year for $1.00 cash in advance. No checks. Strictly to new subscribers.**

---

### SURGICAL HINTS.

In burns about the neighborhood of the joints, keep the limb flexed if the burn is on the extensor side, and extended if the flexor side is affected.

In burns of the face, where the nose is badly affected, it is often a good idea to pass pieces of rubber drainage tubing up the nostrils in order to prevent closure during cicatrization.

In phlegmonous inflammation of the last phalanx it is well to cut down to the bone, but if the middle or first phalanx is affected it is better surgery to avoid going through the sheath of the tendon.

If you can help it, don't operate on a man who is drunk, especially if he appears to be an habitual drunkard. Drunkenness certainly seems to favor the occurrence of sepsis, owing to diminished resistance of the tissues, and shock occurs very readily. Besides this, delirium tremens may come on to complicate matters.

Phimosis and balano-posthitis occasionally occur in adults in whom no venereal disease is at fault. In these cases it is well to examine the urine before operating, for diabetes may be the cause, and in the latter case it is not always safe to operate. Diabetic gangrene of the penis has also been mistaken for phagedenic chancre.

Always give a very guarded prognosis as to the ultimate results in deep phlegmonous processes occurring in the hand. The tendons are often immobilized within their sheaths, the muscles are atrophied, and much of the cellular tissue takes on a cicatricial character. The fingers are, therefore, apt to become stiffened in a curved, claw-like position.

If a patient suffering from urethral stricture has chills every time you pass an instrument, notwithstanding the fact that you are certain that your sounds are perfectly sterile, give up dilatation and cut. The patient will probably have another chill after the operation, but as it is of nervous origin in all probability the danger is very slight, and the subsequent passing of sounds will commonly be accomplished without further trouble.

When tendons of the fingers or toes have been severed, it is often necessary, in order to bring the ends together, to strongly flex or extend the joints, according to whether the flexor or extensor tendons have been injured, and this position should be maintained by the dressing after the suture has been done, in order to relieve any tension upon the severed ends. Passive motion should be made as soon as it is likely that union has well begun.—*Inter. Jour. of Surg.*

---

### THE EARLY DIAGNOSIS OF TUBERCULOSIS.

It is a well recognized fact that the curability of tuberculosis, other conditions being equal, is in direct ratio to the period at which the disease is diagnosticated. Efforts should therefore be directed to determine the diagnosis at the earliest possible stage. Examination of the sputum, though often positive, is frequently in the early doubtful cases negative, and valuable time is lost in the waiting.

Levy and Bruns, in a recent number of the *Deutsche med. Woch-*

*enschrift*, recommend that guinea-pigs be injected with the sputum of suspected cases and killed after several weeks to determine if tuberculosis of the abdominal organs has developed. At times these animals die in a few days from suppurative peritonitis due to the diplococcus pneumoniæ or the streptococcus pyogenes.

B. Fränkel, in the *Berliner klin. Wochenschrift*, advocates the general use of tuberculin in early cases as the most sensitive of all the methods to determine tuberculosis. Although the fiascos, which have followed the therapeutic use of both the old and the new tuberculin, have made many of the profession skeptical about its value and safety even for diagnostic purposes, its undoubted success in accurately determining the presence of bovine tuberculosis makes its employment in obscure cases well worth considering. Where general and local reaction follow the use of tuberculin, tuberculosis can with certainty be said to be present. An increase in temperature of a half degree centigrade indicates a reaction. For several days before the injection of the tuberculin, the ordinary temperature of the patient is determined at intervals of three hours and this is continued during the test. If no reaction follows the first injection of one milligram of tuberculin, then after three or four days five milligrams are injected. If this also is negative, six to ten times the initial dose is employed. If no reaction occurs, tuberculosis is absent. This carefully graded method of using tuberculin can do no harm and failures in diagnosis in the early stage have been very rare. For surgical tuberculosis this method is especially applicable as the local reaction is easily observed.—*Albany Med. Annals.*

---

### SHOCK AND ITS SURGICAL SIGNIFICANCE.

J. H. Rishmiller, Minneapolis (*New York Medical Journal*, March 10 and 17, 1900), summarizes the conclusions reached in a discussion of this subject:

1. Sensory nerve irritation sufficiently powerful to produce exhaustion of the vasomotor center causes a reflex paralysis and consequently a dilatation of the vascular mechanism.

2. Children and aged people with lax fibers and those addicted to alcohol bear a peculiar susceptibility to shock.

3. Hemorrhage is the most pronounced cause, particularly venous, as then the equilibrium of the vasomotor mechanism is too suddenly deranged.

4. Two distinct types are recognized; prostration with indifference, and prostration with excitement.

5. Peritoneal absorption of sepsis invariably terminates fatally through shock before evident manifestations of peritonitis have developed.

6. A subnormal temperature, irregular pulse, superficial respiration, cold and anemic extremities and clammy perspiration, contraindicate an operation.

7. The severity of operative shock largely depends upon the length of time in its performance, and the duration and degree of the anesthesia.

8. Shock may to a large degree be prevented by any counter-irritation applied to the extremities.

9. Brandy per orem and morphine subcutaneously before operating are imperative precautions towards prophylaxis.

10. The main treatment consists in stimulating the vascular system, preserving the animal heat and supplying artificial heat to the body.

11. In acute hemorrhage or other excessive anemia an effusion of normal saline solution is prudently indicated.

---

### TREATMENT OF THE NAVEL.

The chief principles to be observed in the treatment of the navel following birth are, according to A. Ahlfeld (*Centralbl. f. Gyn.*, Nor. 13, p. 337), shortening of the cord to the recognized minimum, touching up the stump and vicinity of the navel with 95 per cent. alcohol, then placing a layer of sterilized cotton which is to remain on for five or six days, only being removed in case of its becoming moist with urine. Especially may it be noted that after the birth of the child the cord is to be cut about 3 to 4 inches from the navel, after carefully tying it off with linen tape. Then, after the bath, the secondary shortening is to be performed. This consists in tying off the cord about two-fifths of an inch from the navel and a little beyond this the cut is made. Now the aforementioned moistening with alcohol is to be done and the sterile cotton wrapped over it. The author never uses silk ligatures for tying off the cord, as it frequently happens that a hematoma of the cord follows a too tightly applied silk thread. The suggestion that a cautery-scissors be used to cut off the cord about one-half inch from the child's abdomen, Ahlfeld scores as being too dangerous in its application.—*Medical News.*

---

### INFANTILE SCURVY.

There are two affections occurring in children for which scurvy is particularly mistaken; acute rheumatism and acute anterior poliomyelitis. There is practically no reason at all why scurvy should be confounded with the latter disease. There is of course, absence of movement in the limbs in both cases, but in anterior poliomyelitis this is due to inability to move them because of palsy; in scurvy the failure of movement is due to pain. This can very easily be decided by passive movements. Scurvy and acute rheumatism are not so easy to differentiate. In scurvy, however, the hemorrhage is not into the joint and not into the epiphysis, but practically always into the diaphysis of the long bones. Blood extravasations occur at times over the tissue and occasionally even over the carpus, but these are rare exceptions. If the bones are protected from motion, it will be found that the joints in scurvy may be freely moved. At times, in scurvy, hemorrhage occurs into the joints, and this may almost hopelessly confound the disease with rheumatism; but these joint hemorrhages are very rare.—*A. Jacobi, Medical News.*

---

### PAIN.

Pain of a neuralgic or drawing character in the neighborhood of the heart, is found as the result of several causes, as a rule, in the following order of frequency:

1. Pain with palpitation of the heart from the accumulation of flatus in the transverse colon just as it turns to descend. Many patients who go to the physician complaining of heart disease suffer only from this condition, due to fermentation in the large bowel. Again, the pain due to gastralgia, or, as it has been called, cardialgia, may be referred to the heart by the patient.

2. Intercostal neuralgia due to debility. In these cases tender spots will often be found, one in the precordium, another in the outer edge of the scapula, and a third on the vertebral column. These are sometimes called "spots of Valleix." In other cases the pain will be due to spinal trouble, anemia, or tight lacing of corsets.

3. Pseudo-angina.

4. True angina.

5. Locomotor ataxia.—*H. A. Hare in Practical Diagnosis.*

---

### THE TREATMENT OF CATARRHAL CONJUNCTIVITIS.

BY MILTON P. CREEL, M.D., CENTRAL CITY, KY.

Either as it appears as a simple catarrhal inflammation of the conjunctiva, affecting one individual or when it is encountered in an epidemic, there is no doubt but that catarrhal conjunctivitis is an affection of great importance. This affection is essentially simple, but if allowed to go along without correct treatment it may terminate in entire loss of vision. However, if the affection be given proper and timely attention it yields with great readiness to treatment.

Either as simple catarrhal conjunctivitis seen in a single individual, or when the affection manifests itself in the epidemic form the treatment is essentially the same. Of course, individual peculiarities in each case make certain indications fitting and even imperative. One thing which a large experience with the disease has taught me is, that prompt and systematic treatment must be instituted in every case. Often patients with strumous diathesis will have chronic conjunctivitis, and persons whose health is poor will also have protracted forms of the affection, with the loss or great impairment of sight, when if proper and timely treatment had been instituted a cure could have been effected within a very short time. In the treatment of catarrhal conjunctivitis there have been many mischievous measures brought to bear.

All and everything which produces irritation will render all the elements in the case worse. We must never employ strong solutions. A lotion composed of 10 grains of sulphate of zinc to an ounce of distilled water will aggravate any case. All lotions must of necessity be mild and soothing.

As a curative means I have come now to rely on what I term the antiseptic treatment. This has been productive of better results in my hands than the old-time remedies.

In carrying out this treatment I first have the nurse to bathe the eyes thoroughly with this antiseptic mixture:

℞ Hydrozone, .................................................... ʒ j

Aqua, q. s. ad .................................................... ℥ iv.

This mixture is used three or four times daily, as the case may appear to demand. Just as often as this mixture has been copiously applied and the eyelids have been dried, I apply, by means of an ordinary glass medicine dropper, two drops of Marchand's Eye Balsam.

This remedy reaches every part of the conjunctiva by the movements of the lids, and it is not irritating; the patient generally makes rapid progress to recovery.

By this treatment I have found my patients to recover in from thirty-six hours to three days. In fact my success has been such that I now rely upon this treatment entirely in this affection.

Four months ago an epidemic of catarrhal conjunctivitis broke out in a boarding-school. I was called and ordered these remedies used on every case that presented itself. The nuns told me that all the cases got well speedily.

Mr. Samuel S., aged 39. This patient had been suffering, as he put it, with "sore eyes" for three days. It was a simple case of catarrhal conjunctivitis, but gave him great discomfort. On the treatment described above he entirely recovered in two days.

Mrs. Laura S., aged 22. This patient thought she had something in her eye, but examination revealed catarrhal conjunctivitis. On this treatment she made a speedy recovery.

These are only two of the several hundred cases treated on the antiseptic principles.—*Medical Summary.*

